# Evaluation of magnetic resonance imaging and deep learning-based synthetic computed tomography for calcified intradural tumors – importance of domain-specific training and validation of synthetic imaging methods for clinical application

**DOI:** 10.1007/s00701-025-06731-0

**Published:** 2025-12-11

**Authors:** Gregor Fischer, Felix C. Stengel, Lorenzo Bertulli, Linda Bättig, Victor E. Staartjes, Tobias Dietrich, Olaf Chan-Hi Kim, Martin N. Stienen

**Affiliations:** 1 Department of Neurosurgery, H-OCH Health Ostschweiz & St. Gallen Medical School, Cantonal Hospital of St. Gallen, St. Gallen, Switzerland; 2 Interdisciplinary Spine Center, H-OCH Health Ostschweiz & St. Gallen Medical School, Cantonal Hospital of St. Gallen, St. Gallen, Switzerland; 3 Machine Intelligence in Clinical Neuroscience & Microsurgical Neuroanatomy (MICN) Laboratory, Department of Neurosurgery, Clinical Neuroscience Center, University Hospital Zurich, University of Zurich, Frauenklinikstrasse 10, CH-8091 Zurich, Switzerland; 4 Department of Radiology and Nuclearmedicin, H-OCH Health Ostschweiz & St. Gallen Medical School, Cantonal Hospital of St. Gallen, St. Gallen, Switzerland

**Keywords:** BoneMRI, Intradural tumor, Meningioma, Calcification, Synthetic imaging, AI-generated images

## Abstract

**Purpose:**

For intradural spinal tumors, information on the degree of calcification is helpful to plan the surgery. Novel deep-learning algorithms allow to generate synthetic computed tomography (CT) images from magnetic resonance imaging (MRI).

**Methods:**

We conducted a prospective observational cohort study, including *n *= 105 patients with spinal pathologies between 07/2022 – 09/2023, to validate the accuracy of BoneMRI (MRIGuidance BV©, Utrecht, the Netherlands). Patients underwent both conventional CT and MRI; synthetic CT images were generated from MRI source data with artificial intelligence (AI). For the scope of this post-hoc analysis, only patients with intradural tumors were selected.

**Results:**

Five patients with intradural tumors of the spine were included (mean age 67.8 years; 4 (80%) female). The tumors were visible on 5/5 conventional CT images (100%), on average 19.6 × 11.6 mm in size and 4/5 (80%) were densely calcified (mean Hounsfield units (HU) 463.6). Although well-visible on the T1w/T2w/BoneMRI source data, none of the tumors showed up (0%) on synthetic CT. Visible tumor dimensions were 0 mm in both axial (*p* < 0.001) and sagittal planes (*p* = 0.017), with an average density of 20.9 HUs (*p* = 0.034).

**Conclusions:**

BoneMRI generated synthetic CT is a promising, radiation-free alternative to conventional CT. Intradural tumors – even those with dense calcifications – were not visualized by synthetic CT images, highlighting that this novel technology is currently not able to capture lesions outside its main scope. Our analysis demonstrates powerfully that synthetic imaging must be cautiously applied to populations for which it was developed and validated, and that any extrapolation can be clinically misleading.

**Supplementary Information:**

The online version contains supplementary material available at 10.1007/s00701-025-06731-0.

## Introduction

Intradural extramedullary tumors of the spine (IDEMs) constitute a group of usually benign neoplasms, which require surgical resection if symptomatic and/or progressive in size [1, 6, 35]. Treatment is typically performed under microscopic magnification, using minimally invasive approaches and sophisticated intraoperative tools to lower the surgical risk and improve outcome [15]. The degree of a lesion’s calcification – hence density – is one of the factors determining the choice of surgical approach, resection technique and surgical tools, which is why this information is essential for planning the case. Moreover, the degree of calcification has been shown to correlate with surgical morbidity and postoperative outcome [29].

While intradural tumors are best detected and their relationship to the nerve structures visualized by magnetic resonance imaging (MRI), computed tomography (CT) is additionally requested nowadays to determine the degree of intra-tumoral calcifications and potential osseous involvement. However, CT requires ionizing radiation, which increases the risk for carcinogenesis, which is particularly important to consider when dealing with pediatric patients or younger adults [2–4]. Recently, progress in the application of artificial intelligence (AI) in healthcare has led to the development of accurate synthetic CT (sCT) images from MRI sequences [8, 12, 17, 21, 32, 37], presenting a potentially interesting alternative for many clinical scenarios today. BoneMRI (MRIGuidance BV©, Utrecht, the Netherlands) was developed to illustrate the bony anatomy of the spine and pelvis [8, 17, 21, 32, 37]. The algorithm was trained on a wide range of pathologies including degenerative disease, deformities, rheumatological disease and a limited number and type of malignancies, however not on intradural tumors.

So far, no clinical studies have been performed on the performance and diagnostic validity of BoneMRI for less prevalent diseases [36], such as intradural tumorous lesions. By evaluating the performance of BoneMRI for patients with intradural tumors including importantly highly calcified lesions – a subset not part of the training data scope of the algorithm – we aim to elucidate the importance of avoiding extrapolation when applying synthetic imaging in the clinical setting.

## Methods and materials

### Type of study & ethical considerations

A prospective, observational multi-center study, approved by the local ethics committees of each contributing center, was conducted between 09/2022 and 03/2024 to qualitatively and quantitatively evaluate the diagnostic validity of sCT [12]. Written informed consent was obtained from all participants. At the main site in Switzerland (Cantonal Hospital St.Gallen, University of St.Gallen), some patients with intradural tumors were included in the study, which now serve as population for this post-hoc subgroup analysis.

A research grant was paid from MRIGuidance BV© (Utrecht, the Netherlands) to the participating hospitals to cover the study expenses only, but none of the authors report any personal conflicts of interest. All authors had full control of inclusion of any data and information submitted for publication.

### Objectives

To evaluate, whether BoneMRI generated sCT depicts calcified intradural tumors, despite not having trained the AI-algorithm on this type of lesion. Moreover, we discuss why the knowledge on the degree of calcification is valuable when treating these types of tumors and provide considerations and future perspectives regarding AI-generated images.

### Study cohort and patient inclusion

Patients aged between 18 and 80 years with a diagnosis of an intradural tumorous lesion, admitted to the Cantonal Hospital of St.Gallen (University of St.Gallen) were enrolled. Exclusion criteria were 1) pregnancy, 2) contraindications to MRI or CT, 3) osteosynthetic material within the spinal segment of interest and 4) failure to complete both imaging examinations. Both MRI and CT data were obtained within a short, usually 24-h interval.

### CT protocol

All patients underwent a non-contrast spine CT-protocol (Somatom Definition FLASH, Siemens Healthineer) using collimation with 0,6 mm pitch factor 0,6, rotation time 0,5 sTube voltage reference of 120 kV, quality reference 200 mAs, image reconstruction: 1 mm slice thickness, increment 0,7, Kernel i70f.

### MRI protocol

The usual MRI examination was complemented by an additional 4-min 3D radiofrequency-spoiled T1-weighted multiple gradient echo (T1w-MGE) sequence (2 echoes; repetition time (TR) 7 ms, echo time (TE): TE1 1.1–2.1 ms, TE2 2.2–4.2 ms; field of view (FOV) 250 × 250 × 90 mm; reconstructed voxel size 0.74 × 0.74 × 0.9 mm, acquisition time 3 min and 53 s) to generate sCT reconstructions (BoneMRI) [8, 17, 21, 32, 37]. A commonly available T1w-MGE was supplemented to a standard 1.5 T or 3 T spine MRI acquisition protocol, with T2w sequences in sagittal and STIR in coronal planes. Various MRI systems including Siemens Healthineers MAGNETOM Skyra 3 T (3D fld2, FOV 280 mm, 4:40 min, slice thickness 0.8 mm, 120 slices) and General Electric HealthCare (SIGNA Hero 3 T) with a TR, FOV 250 × 250 × 90 mm with reconstructed voxel size = 0.74 × 0.74 × 0.9 mm^3^), were used.

### BoneMRI—synthetic CT (sCT) reconstruction

BoneMRI reconstructions or sCT’s were generated from T1w-MGE MRI using a deep learning-based image synthesis method, with commercially available software (BoneMRI v1.4.0alpha, MRIGuidance BV©, Utrecht, the Netherlands). This method exploits local spatial contextual information from multiecho data to reconstruct the underlying bony structures, following an a priori training protocol using paired MRI and CT data [14]. The resulting sCT images not only provide qualitative illustration of osseous structures, but additionally offer quantitative information related to radiodensity as expressed by HUs. In this study, reconstructions were performed off-site at MRIguidance using a Horos PACS (Version 3.3.6 (LGPL-3.0).

### Image analysis

**Fig. 1:**
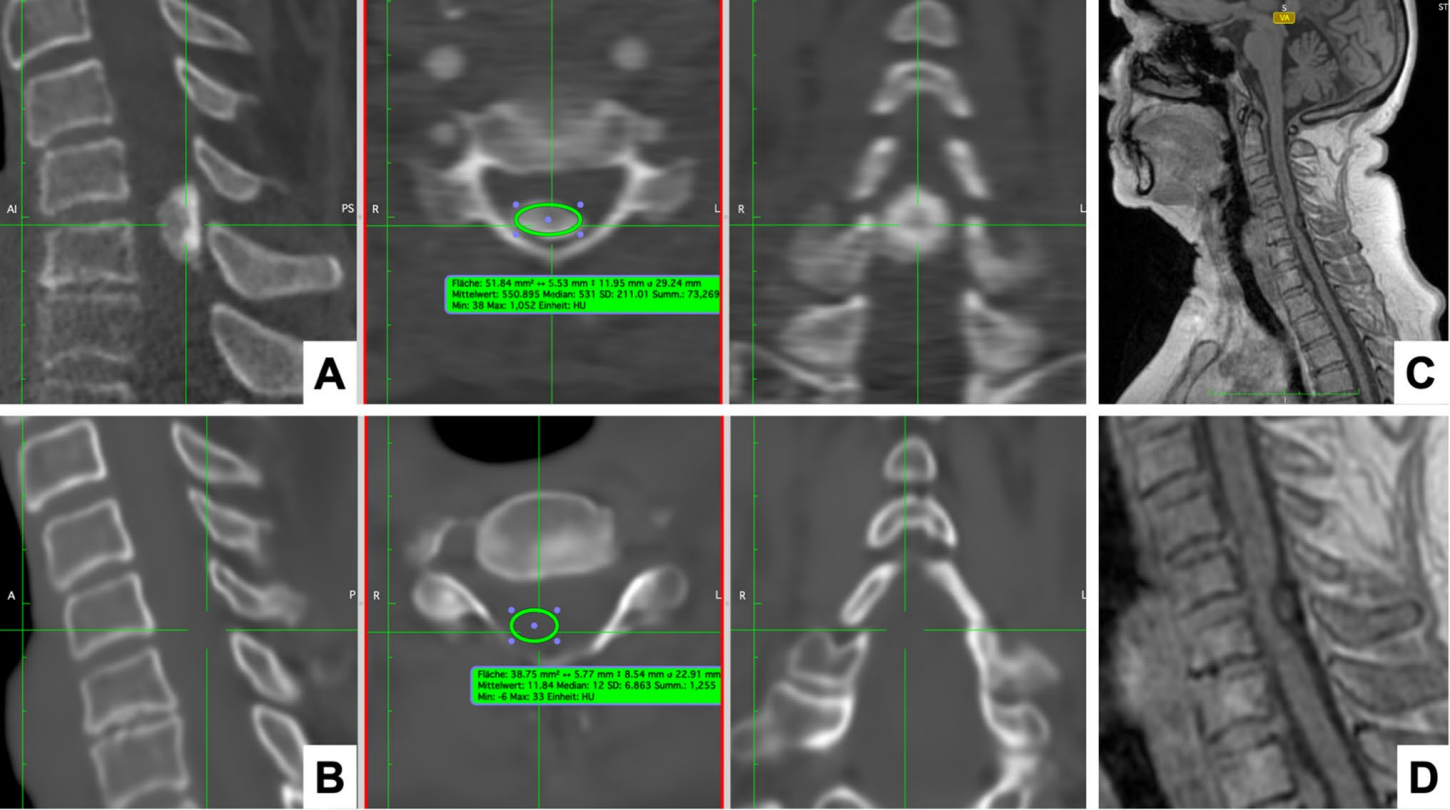
Case vignette of a 71-year-old female patient with a densely calcified intradural meningioma (mean HUs 551). **A** In the conventional CT image, the densely calcified tumor is well visible, the dimensions measured were 13.8 mm (axial) x 16 mm (sagittal) and mean HUs of 550.8 in the axial view. **B** On the synthetic CT image, the tumor was not visible (0 × 0 mm), and the mean HUs of the tumor region were 11.8. **C-D** On the source image (3D radiofrequency-spoiled T1-weighted multiple gradient echo (T1w-MGE) sequence), the tumor can be seen

A panel of two spine surgeons (G.F. and M.N.S. with 5 and 13 years of experience in spine surgery, respectively) independently reviewed the images for diagnostic accuracy and lesion characterization. Imaging analysis was performed using Xero Viewer (Version 1.0.0.R812, AGFA HealthCare) and Horos (Version 3.3.6, OsiriX). T2-weighted and STIR MRI Sequences, sCT, and CT scans were displayed to assessors for evaluation of intradural tumors. All physicians were blinded to further clinical data and other images during the evaluation process. Clinically relevant radiological features, such as visibility of the tumor and tumor morphology were assessed and compared between both imaging modalities using geometrical parameters such as maximal axial and sagittal diameter, as well as density in Hounsfield Units (HUs; Fig. 1). The region of interest (ROI) for the HU measurements in sCT images was set to the area where the intradural tumor was evident in the MRI source sequences.

### Statistical analysis

All statistical analyses were performed using Stata SE (StataCorp LLC, College Station, TX, USA) v18.0 software for Mac. Descriptive statistics included means, standard deviations (SDs), ranges, and proportions. Chi-square and student’s t-tests were applied for group comparisons. An alpha level of *p* < 0.05 was considered statistically significant.

## Results

### Patient cohort

A total of five patients (four females; 80%) with intradural tumors and a mean age of 67.8 years (SD 12.7) were included. Their mean body mass index was 27.0 kg/m^2^ (SD 2.7); one smoker (20%), ASA grade II (*n* = 2) and III (*n *= 3), Charlson comorbidity index 0 (*n* = 1), 2 (*n* = 2), 3 (*n* = 1) & 5 (*n* = 1). The spinal segments involved were C5/6, T1/2, T4/5, T5-7 and T8/9 (each *n* = 1). Three patients had clinical signs of myelopathy (60%); one had motor deficits (20%) underneath the spinal level involved.

Four patients underwent surgical treatment; one elderly patient without clinical symptoms and a small, non-compressive tumor was treated conservatively. Surgery took 218 min on average (SD 56.7, range 170–285 min) with a mean estimated blood loss of 150 ml (SD 70.7). In two patients a unilateral laminectomy sparing the posterior tension band was chosen; in the remaining two the approach was laminoplasty. Mean HU of the tumor tended to be higher in patients treated by laminoplasty (766 vs. 260 HU, *p* = 0.129). All patients were operated under continuous neuromonitoring (motor and somato-sensory evoked potentials), with use of intraoperative ultrasound (bk5000 with Hockey Stick Transducer X18L5s; BK Medical, Burlington, MA) and an ultrasonic aspiration device (Sonopet iQ with 12 cm iQ micro tip for soft tumors, iQ serrated tip for partially calcified tumors & iQ micro claw for densely calcified tumors; Stryker Corporation, Kalamazoo, MI). Gross total resection was achieved in all four patients (100%). Histopathological workup revealed meningioma (CNS WHO grade 1) in all four resected patients.

Length of hospitalization was 8.3 days (SD 4.7; range 5–15 days). One patient undergoing cervical laminoplasty experienced excessive and prolonged approach-related neck pain; no other complications were observed. Length of hospitalization tended to be higher in patients after laminoplasty (11.5 days, SD 4.9), compared to unilateral laminectomy (5.0 days, SD 0, *p* = 0.204). The clinical outcome was excellent in all patients at 90 days and 12 months (100%) without any neurological deficits, residual or recurrent tumor in follow-up imaging.

### Visualization of tumor on imaging

The intradural tumors were visible in 100% of CT images (*n* = 5; well-visible in *n *= 4/5 (80%)) and 0% of sCT images (*n* = 0), while being visible on the T1w/T2w/BoneMRI source data in 100% of cases. The tumor dimensions were 11.6 vs. 0 mm (*p* < 0.001) on axial and 19.6 vs. 0 mm on sagittal imaging (*p* = 0.017). Mean HU of the tumor region were 463.6 on CT vs. 20.9 on sCT (*p *= 0.034) in the ROI. The key images of all five patients are displayed as Supplemental Figs. 1–5. Synthetic CT did not display any indirect signs of intradural tumor growth, including spinal cord displacement (visible on MRI source data), bone remodeling or dural tail signs.

## Discussion

The group of IDEMs comprises meningiomas (20–30%), schwannomas (15–50%), followed by neurofibromas and further, less common types of neoplasms [20, 35]. In general, IDEMs are usually benign lesions that grow gradually and eventually induce neurological symptoms resulting from spinal cord or nerve compression. If symptomatic or progressive in size, microsurgical resection is recommended, as it represents an effective and safe treatment option with favorable outcome and improvements in health-related quality of life in the vast majority of patients [1, 11, 29, 34]. In this series, five patients with intradural meningiomas and various degrees of intra-tumoral calcifications were reviewed, applying a novel MRI-based technology to generate synthetic CT with the help of AI. The intra-tumoral calcification was not visualized by this technology, which is important to acknowledge and triggers further considerations on the use of AI-generated synthetic imaging in modern medicine.

### Surgical technique, depending on degree of calcification

**Fig. 2:**
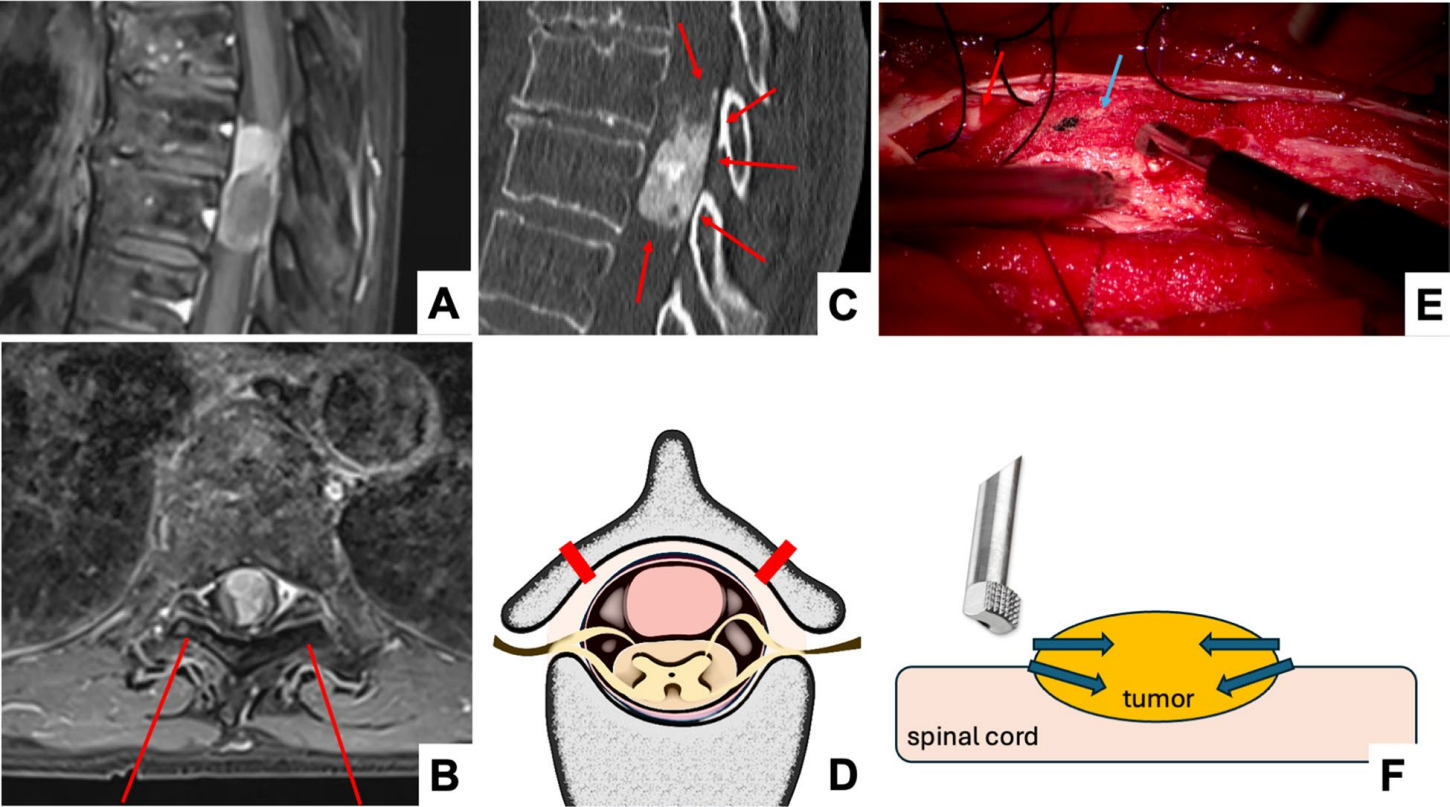
Example of a densely calcified intradural meningioma in a 68-year-old female patient with a calcified, intradural tumorous lesion at the T8/9 level. The patient was myelopathic and presented with motor deficits of both lower extremities to our outpatient clinic. **A** Sagittal contrast-enhanced T1 MRI-sequence showing the maximal cranio-caudal extension of the mass. **B** Axial contrast-enhanced T1 MRI-sequence showing the degree of spinal cord deviation and compression, as well as the bilateral approach chosen for the laminoplasty (red lines). **C** Sagittal plain CT-scan, outlining the tumor margins well (red arrows). **D** Illustration (axial view) of the bilateral cuts for the laminoplasty, to achieve a good visualization of both lateral borders of the tumor. **E** Intraoperative microscopic image during tumor resection. The spinal cord (red arrow) is protected by cottonoids (blue arrow) while the bone-cutting ultrasonic aspirator tip is used. **F** Illustration of the surgical technique for “hard tumors”, using a bone-cutting 90° ultrasonic aspirator to start peripherally and reduce the tumor size without inducing any vertically oriented pressure on the underlying spinal cord

Densely calcified meningiomas are physically hard, making them more challenging to resect compared to soft tumors with little or no intra-lesional calcification. For densely calcified tumors, especially for those extending to the contralateral side or those situated posterior to the dentate ligament, a midline approach offers the advantage that the tumor margins can be controlled from the beginning of the resection. This enables the use of a 90° angled bone-cutting tip of the aspiration device, which transmits the forces acting upon the tumor in a horizontal rather than vertical fashion (Fig. 2; Supplemental Video 1). In contrast to soft tumors, calcified lesions cannot absorb the vibrations and pressure but forward it directly to the underlying spinal cord, which may induce myelopathic changes and injure the neural tissue. Therefore, no downward pressure is allowed, which is more difficult to avoid when choosing a unilateral approach and beginning with intra-lesional debulking. Contrary to the softer tumors, resecting densely calcified tumors is started at the periphery working towards the middle (Fig. 2). To facilitate this, midline approaches include classic laminectomy without, or laminoplasty with reconstruction of the spinal canal roofing. In a systematic review and meta-analysis comparing both approach types for intradural tumors, Byvaltsev et al. found that laminoplasty was on average 17min shorter (not significant) and led to a shorter mean hospital stay by about 3.5 days (*p *< 0.001) [5]. While the odds ratio to experience any complication after laminoplasty vs. laminectomy was similar (OR 0.83, 95% CI 0.50–1.38), the likelihood to develop a kyphotic deformity during follow-up was in favor of laminoplasty (OR 0.47, 95% CI 0.27–0.84) [5]. At our center, we intend to recreate the posterior roof by laminoplasty in every patient that is selected for bilateral exposure.

**Fig. 3:**
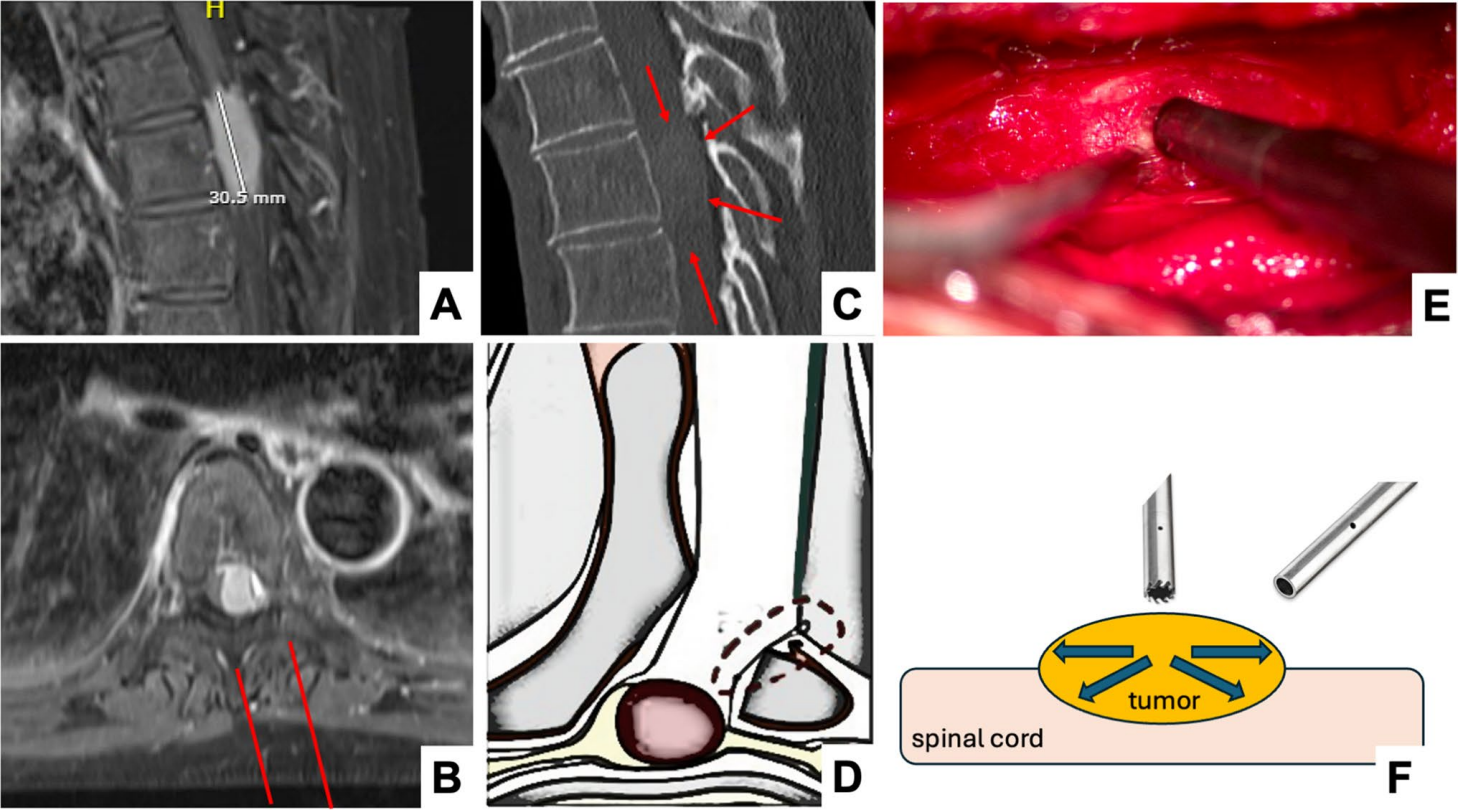
Example of a barely calcified intradural meningioma (mean HUs 113) at the T5-7 level. **A** Sagittal contrast-enhanced T1 MRI-sequence showing the maximal cranio-caudal extension of the mass. **B** Axial contrast-enhanced T1 MRI-sequence showing the degree of spinal cord deviation and compression, as well as the unilateral sub-periosteal approach chosen for the microsurgical resection (red lines). **C** Sagittal plain CT-scan, outlining the tumor margins slightly (red arrows). **D** Illustration of the “lateral dural tacking method” to maximize the microscopic visualization of the intradural space, according to Yeo et al., 2011 (axial view). **E** Intraoperative microscopic image during internal tumor debulking using the micro-tip of the ultrasonic aspirator. **F** Illustration of the surgical technique for “soft tumors”, using an ultrasonic aspirator to centrally debulk the tumor before dissecting the peripheral parts off the spinal cord. Depending on the degree of calcification, small “micro-tips” or larger tips with more bone-cutting potential may be used

On the contrary, soft extramedullary tumors can be approached using a unilateral (hemi-)laminectomy approach with “lateral dural tacking method” to maximize the microscopic visualization of the intradural space [38]. The use of an ultrasonic aspiration device is often helpful to internally debulk the tumor before dissecting it off the spinal cord and nerve structures [15]. The working direction is hence from the center to the more peripheral parts (Fig. 3). The straight micro-tip of the aspiration device may induce some vibrations and downward pressure, acting on but usually not jeopardizing the spinal cord when tumors are soft (Fig. 3; Supplemental Video 2). Even lesions situated anterior to the dentate ligament and those with a cross-sectional occupying ratio of > 90% can safely be removed by this technique [38]. In a comparative retrospective series of *n *= 70 IDEMs, Pompili et al. showed that the unilateral approach did not increase the risk for residual tumor mass, while lowering the risk for postoperative instability [26]. Dobran et al. showed in their series of *n* = 40 IDEMs that hemilaminectomy (*n *= 13) versus laminectomy (*n* = 27) lead to less surgical time (90 vs. 160 min, *p* < 0.001), less time spent in bed postoperatively (48 vs. 72 h, *p* < 0.001), shorter hospitalization (7 vs. 12 days, p < 0.001) and less approach-related pain (visual analog scale 2.5 vs. 4.6, *p* < 0.001), while providing a similar extent of resection [9]. This compares well with our observations in this series, indicating more postoperative pain and longer hospitalization in the patients treated with bilateral laminoplasty compared to the unilateral approach. Fortunately, the fraction of patients with benign IDEMs requiring long-term opioid medication for approach-related pain is small [19].

As the surgical approach and resection technique applied differs in hard versus soft intradural tumors, it is important to understand the degree of intra-tumoral calcifications preoperatively when planning the surgery.

### Implications for practice

Intradural tumors are usually diagnosed on MRI. Intra-tumoral calcifications can sometimes be appreciated on MRI using gradient echo or susceptibility-weighted imaging sequences (GRE/SWI), but for the reasons outlined above, additional CT images are nowadays requested before surgery. Further reasons for adding a CT scan to the preoperative diagnostic panel include the study of the detailed bony anatomy of the lamina and pedicles (to tailor the approach) and to check for calcification within the “dural tail”, an infiltration and thickening of the peritumoral dura, which may contain vital tumor cells and should ideally be removed [30].

Applying modern technology to generate reliable CT-like images from MRI sequences in this setting would streamline the preoperative workflow, would save both time and resources (money, equipment, required staff), and avoid exposing the patient to radiation. The synthetic CT generated by the BoneMRI technology has been shown to be highly accurate and reliable to depict the anatomy of the spine and pelvis [8, 17, 21, 32, 37], but it currently fails to visualize intradural lesions. Although possibly explainable from an engineering perspective (intradural lesions were likely not part of the training dataset), we were still surprised about the complete ignorance of the software for even densely calcified lesions, as they are easily visible on the source data used to generate the synthetic CT images (Supplemental Figs. 1–5). This demonstrates a powerful point in the distinction of synthetic versus “real”/physical imaging: Whereas in physical imaging, each voxel or pixel is based on a physical measurement (e.g. radiodensity for x-ray/CT), synthetic imaging methods make up part or all of an image to supply more complex visualizations from simple data. The latter is thus, as with all machine learning methods, vastly dependent on the scope and distribution of data seen during training. Thus, this is a stark contrast to the results demonstrated herein for conventional CT: calcifications are easily visible (Supplemental Figs. 1–5). Even extremely rare entities such as primary bone tumors can be accurately visualized by a conventional spiral CT, where the image is completely based on physical measurements – whereas synthetic imaging methods such as BoneMRI are powerful tools only in the applications they were developed for and rigorously validated in.

Our findings point out some relevant implications regarding the globally rising use of AI-generated images in healthcare [27]. Use of AI in medical imaging enables rapid and accurate detection of abnormalities, speeds up the interpretation of complex images and improves early detection of diseases, ultimately delivering better patient outcomes. AI continues to shape the future of healthcare in profound and positive ways [25]. However, studies such as ours point out that currently there are limitations to the use of AI in healthcare. Algorithms may fail to display lesions that they were not trained to identify, which could lead to misdiagnoses or missed diagnoses. The fundamental limitation underlying the synthetic CT's sensitivity to detect rare pathologies stem from training datasets predominantly containing healthy bone structures and common degenerative conditions. Especially when dealing with exceptionally rare and unexpected findings that are not displayed [36], physicians blindly trusting AI-generated images might be unpleasantly surprised by prolonging the diagnostic process. Current deep learning algorithms lack sufficient representation of rare pathologies, particularly calcified tumors and intradural lesions [10, 31]. This creates a systematic bias where AI cannot recognize patterns it has never encountered. The reported difficulty of measuring bone mineral density and detecting osteoporosis provides a further cautionary example. Despite being a common condition, sCT showed reduced diagnostic accuracy for osteoporosis because training datasets were underrepresented in terms of osteoporotic bone changes [18]. If a prevalent condition like osteoporosis is challenging to display, rare calcified intradural tumors face even greater detection barriers. It seems vital for image-generating software developers and clinicians applying new technology to cross-validate novel products on very diverse datasets, to appreciate the strengths, but to also understand the inherent limitations of their product. Creating CT-like images from MRI source data is primarily used to diagnose diseases and injuries of the skeleton today [8, 12, 17, 21, 32, 37], but their therapeutic application beyond radiotherapy planning – e.g., for spinal navigation – is currently evaluated [7, 22, 28].

In our opinion, use of novel technology optimizing healthcare delivery should be embraced, but caution is needed to not overestimate its capabilities. Future advances may address these limitations through expanded training datasets, specialized MRI sequences, and uncertainty-aware algorithms, but the current version cannot replace CT for rare conditions like intradural calcified tumors. For technology generating CT-like images of the spine, it would be ideal to allow the identification and accurate display of all possible types of calcified lesions, including but not limited to calcified thoracic disc herniations [13], ossification of the posterior longitudinal ligament (OPLL) [14], calcified synovial cysts of the zygapophyseal joints [16], ossification of the ligamentum flavum [24], calcifying pseudoneoplasms of the neuraxis (CAPNON) [33], or spinal arterio-venous malformations [23]. Reports such as ours remind clinicians to be weary of extrapolation and give the software developers more insights, which can be used to improve the product in future versions by adding specific pathologies to the training dataset. Specifically, the development of datasets including variably calcified lesions, ranging from densely mineralized meningiomas to subtly calcified pathologies, is essential for advancing sCT technology beyond its current limitations.

## Conclusion

Understanding the degree of intra-lesional calcification and density is relevant to tailor the microsurgical approach and to choose the required technological aids, such as ultrasonic aspiration devices. Intradural tumors – even those with dense calcifications – were not visualized by synthetic CT images, highlighting that this novel technology is not able to capture lesions outside its main scope. Our analysis demonstrates powerfully that synthetic imaging should be cautiously applied to populations for which it was developed and validated, and that any extrapolation can be clinically misleading. In future versions, after having trained and validated the algorithm with more diverse datasets e.g. including intraspinal calcified masses, applications of synthetic imaging can potentially be expanded while retaining high reliability.

## Supplementary Information

Below is the link to the electronic supplementary material.

ESM 1 Supplementary Material 1 (PDF 7.52 MB)

ESM 2 Supplementary Material 2: (1:15 min): Example of the technique used to resect densely calcified intradural meningiomas. After bilateral exposure using laminoplasty, the lateral tumor borders are identified first. A bone-cutting tip of an ultrasonic aspiration device is helpful to reduce the tumor size in a horizontal fashion, making sure no vertical pressure is transmitted onto the spinal cord. Instruments are used for counter-pressure throughout the resection (working direction peripheral – central). The final tumor masses can be dissected off the spinal cord. (PDF 68.1 MB)

ESM 3 Supplementary Material 3: (1:41 min): Example of the technique used to resect soft, barely calcified spinal meningiomas. After unilateral exposure via hemilaminectomy, lateral tack-up sutures are performed to maximize the visualization of the intradural space. After understanding the tumor-nerve interface, a small tip of an ultrasonic aspiration device is used to centrally debulk the tumor mass, before dissecting the remaining parts off the spinal cord (working direction central – peripheral). (PDF 568 MB)

## Data Availability

The data underlying this analysis are available from the corresponding author upon reasonable request.

## Abbreviations

AI: Artificial intelligence
CAPNON: Calcifying pseudoneoplasms of the neuraxis
CT: Computed tomography
IDEM: Intradural extramedullary tumor of the spine
HU: Hounsfield units
MRI: Magnetic resonance imaging
OPLL: Ossification of the posterior longitudinal ligament
OR: Odds ratio
ROI: Region of interest
T1w-MGE: T1-weighted multiple gradient echo
TE: Echo time
TR: Repetition time
sCT: Synthetic CT
SD: Standard deviation
SWI: Susceptibility-weighted imaging

## References

[1] Ali AMS, Mustafa MA, Ali OME, Gillespie CS, Richardson GM, Clark S, Wilby MJ, Millward CP, Srikandarajah N (2024) Patient-reported outcomes in primary spinal intradural tumours: a systematic review. Spinal Cord 62:275–284. 10.1038/s41393-024-00987-6 38589551 10.1038/s41393-024-00987-6 PMC11199135

[2] Amis ES Jr., Butler PF, Applegate KE, Birnbaum SB, Brateman LF, Hevezi JM, Mettler FA, Morin RL, Pentecost MJ, Smith GG, Strauss KJ, Zeman RK, American College of R (2007) American College of Radiology white paper on radiation dose in medicine. J Am Coll Radiol 4:272–284. 10.1016/j.jacr.2007.03.002 17467608 10.1016/j.jacr.2007.03.002

[3] Brenner D, Elliston C, Hall E, Berdon W (2001) Estimated risks of radiation-induced fatal cancer from pediatric CT. AJR Am J Roentgenol 176:289–296. 10.2214/ajr.176.2.1760289 11159059 10.2214/ajr.176.2.1760289

[4] Brenner DJ, Hall EJ (2007) Computed tomography–an increasing source of radiation exposure. N Engl J Med 357:2277–2284. 10.1056/NEJMra072149 18046031 10.1056/NEJMra072149

[5] Byvaltsev V, Polkin R, Kalinin A, Kravtsov M, Belykh E, Shepelev V, Satardinova E, Manukovsky V, Riew KD (2023) Laminoplasty versus laminectomy in the treatment of primary spinal cord tumors in adult patients: a systematic review and meta-analysis of observational studies. Asian Spine J 17:595–609. 10.31616/asj.2022.0184 36717092 10.31616/asj.2022.0184 PMC10300886

[6] Dang DD, Mugge LA, Awan OK, Gong AD, Fanous AA (2024) Spinal Meningiomas: A Comprehensive Review and Update on Advancements in Molecular Characterization, Diagnostics, Surgical Approach and Technology, and Alternative Therapies. Cancers (Basel) 16. 10.3390/cancers16071426 10.3390/cancers16071426 PMC11011121 38611105

[7] Davidar AD, Judy BF, Hersh AM, Weber-Levine C, Alomari S, Menta AK, Jiang K, Bhimreddy M, Hussain M, Crawford NR, Khan M, Gong G, Theodore N (2023) Robot-assisted screw fixation in a cadaver utilizing magnetic resonance imaging-based synthetic computed tomography: toward radiation-free spine surgery. Illustrative case. J Neurosurg Case Lessons 6. 10.3171/CASE23120 10.3171/CASE23120 PMC10555644 37458340

[8] Di Dier K, Laloo F, Van Den Berghe T, Vereecke E, Jaremko J, Chen M, Jans L (2025) Spondyloarthritis endgame: MRI versus BoneMRI in sacroiliitis. Skeletal Radiol. 10.1007/s00256-025-04921-6 40140065 10.1007/s00256-025-04921-6

[9] Dobran M, Paracino R, Nasi D, Aiudi D, Capece M, Carrassi E, Lattanzi S, Rienzo AD, Iacoangeli M (2021) Laminectomy versus unilateral hemilaminectomy for the removal of intraspinal schwannoma: experience of a single institution and review of literature. J Neurol Surg A Cent Eur Neurosurg 82:552–555. 10.1055/s-0041-1722968 33845505 10.1055/s-0041-1722968

[10] Edmund JM, Nyholm T (2017) A review of substitute CT generation for MRI-only radiation therapy. Radiat Oncol 12:28. 10.1186/s13014-016-0747-y 28126030 10.1186/s13014-016-0747-y PMC5270229

[11] El-Hajj VG, Pettersson-Segerlind J, Fletcher-Sandersjoo A, Edstrom E, Elmi-Terander A (2022) Current knowledge on spinal meningiomas-surgical treatment, complications, and outcomes: a systematic review and meta-analysis (Part 2). Cancers (Basel) 14. 10.3390/cancers14246221 10.3390/cancers14246221 PMC9777510 36551706

[12] Fischer G, Schlosser TPC, Dietrich TJ, Kim OC, Zdravkovic V, Martens B, Fehlings MG, Jans L, Vereecke E, Stienen MN, Hejrati N (2025) Radiological evaluation and clinical implications of deep learning- and MRI-based synthetic CT for the assessment of cervical spine injuries. Eur Radiol. 10.1007/s00330-025-11644-8 40335658 10.1007/s00330-025-11644-8

[13] Gong M, Liu G, Guan Q, Li L, Xing F, Xiang Z (2018) Surgery for giant calcified herniated thoracic discs: a systematic review. World Neurosurg 118:109–117. 10.1016/j.wneu.2018.06.232 30017754 10.1016/j.wneu.2018.06.232

[14] Head J, Rymarczuk G, Stricsek G, Velagapudi L, Maulucci C, Hoelscher C, Harrop J (2019) Ossification of the posterior longitudinal ligament: surgical approaches and associated complications. Neurospine 16:517–529. 10.14245/ns.1938222.111 31607083 10.14245/ns.1938222.111 PMC6790740

[15] Henzi S, Krayenbuhl N, Bozinov O, Regli L, Stienen MN (2019) Ultrasonic aspiration in neurosurgery: comparative analysis of complications and outcome for three commonly used models. Acta Neurochir (Wien) 161:2073–2082. 10.1007/s00701-019-04021-0 31377957 10.1007/s00701-019-04021-0 PMC6739453

[16] Huang KT, Owens TR, Wang TS, Moreno JR, Bagley JH, Bagley CA (2014) Giant, completely calcified lumbar juxtafacet cyst: report of an unusual case. Glob Spine J 4:175–178. 10.1055/s-0033-1363591 10.1055/s-0033-1363591 PMC4111943 25083359

[17] Iwasaka-Neder J, Bedoya MA, Connors J, Warfield S, Bixby SD (2024) Morphometric and clinical comparison of MRI-based synthetic CT to conventional CT of the hip in children. Pediatr Radiol 54:743–757. 10.1007/s00247-024-05888-7 38421417 10.1007/s00247-024-05888-7

[18] Jiang Z, Zhu Y, Wang W, Li Z, Li Y, Zhang M (2025) Evaluation of MRI-based synthetic CT for lumbar degenerative disease: a comparison with CT. Sci Rep 15:20548. 10.1038/s41598-025-05399-x 40596109 10.1038/s41598-025-05399-x PMC12219671

[19] Jin MC, Ho AL, Feng AY, Zhang Y, Staartjes VE, Stienen MN, Han SS, Veeravagu A, Ratliff JK, Desai AM (2021) Predictive modeling of long-term opioid and benzodiazepine use after intradural tumor resection. Spine J 21:1687–1699. 10.1016/j.spinee.2020.10.010 33065272 10.1016/j.spinee.2020.10.010

[20] Koeller KK, Shih RY (2019) Intradural extramedullary spinal neoplasms: radiologic-pathologic correlation. Radiographics 39:468–490. 10.1148/rg.2019180200 30844353 10.1148/rg.2019180200

[21] Krabbe S, Moller JM, Hadsbjerg AEF, Ewald A, Hangaard S, Pedersen SJ, Ostergaard M (2024) Detection of structural lesions of the sacroiliac joints in patients with spondyloarthritis: a comparison of T1-weighted 3D spoiled gradient echo MRI and MRI-based synthetic CT versus T1-weighted turbo spin echo MRI. Skeletal Radiol 53:2459–2468. 10.1007/s00256-024-04669-5 38592521 10.1007/s00256-024-04669-5 PMC11411003

[22] Lafranca P, Rommelspacher Y, Walter S, Muijs S, Van Der Velden T, Shcherbakova Y, Castelein R, Ito K, Seevinck P, Schlösser T (2024) The safety and accuracy of radiation-free spinal navigation using an ultrashort, scoliosis-specific BoneMRI-protocol compared to CT. Brain Spine 4:103170 10.1007/s00586-025-09151-x PMC12858448 40691585

[23] Liu PC, Huang CC, Chen CL (2023) Spinal arteriovenous malformation with a calcified nodule: illustrative case. J Neurosurg Case Lessons 6. 10.3171/CASE23260 10.3171/CASE23260 PMC10555574 37773758

[24] Martines J, Martines BMR, Araujo JAB, Pinto LEA, de Castro CC (2012) Calcification of the ligamentum flavum in the thoracolumbar spine: an unusual cause of compressive myelopathy. Autops Case Rep 2:25–29. 10.4322/acr.2012.013 31528568 10.4322/acr.2012.013 PMC6735550

[25] Pinto-Coelho L (2023) How Artificial Intelligence Is Shaping Medical Imaging Technology: a survey of innovations and applications. Bioengineering (Basel) 10. 10.3390/bioengineering10121435 10.3390/bioengineering10121435 PMC10740686 38136026

[26] Pompili A, Caroli F, Crispo F, Giovannetti M, Raus L, Vidiri A, Telera S (2016) Unilateral laminectomy approach for the removal of spinal meningiomas and schwannomas: impact on pain, spinal stability, and neurologic results. World Neurosurg 85:282–291. 10.1016/j.wneu.2015.09.099 26475380 10.1016/j.wneu.2015.09.099

[27] Reddy S (2024) Generative AI in healthcare: an implementation science informed translational path on application, integration and governance. Implement Sci 19:27. 10.1186/s13012-024-01357-9 38491544 10.1186/s13012-024-01357-9 PMC10941464

[28] Rommelspacher Y, Schulte AP, Tanner S, Schellhammer F, Kling S, Seevinck P, Girones Sanguesa M, Strauss AC (2025) Evaluation of MRI technologies for surgical spine planning and navigation. Eur Spine J 34:1447–1454. 10.1007/s00586-025-08703-5 39956884 10.1007/s00586-025-08703-5

[29] Ruggeri AG, Fazzolari B, Colistra D, Cappelletti M, Marotta N, Delfini R (2017) Calcified spinal meningiomas. World Neurosurg 102:406–412. 10.1016/j.wneu.2017.03.045 28323183 10.1016/j.wneu.2017.03.045

[30] Sotoudeh H, Yazdi HR (2010) A review on dural tail sign. World J Radiol 2:188–192. 10.4329/wjr.v2.i5.188 21161034 10.4329/wjr.v2.i5.188 PMC2999017

[31] Spadea MF, Maspero M, Zaffino P, Seco J (2021) Deep learning based synthetic-CT generation in radiotherapy and PET: a review. Med Phys 48:6537–6566. 10.1002/mp.15150 34407209 10.1002/mp.15150

[32] Staartjes VE, Seevinck PR, Vandertop WP, van Stralen M, Schroder ML (2021) Magnetic resonance imaging-based synthetic computed tomography of the lumbar spine for surgical planning: a clinical proof-of-concept. Neurosurg Focus 50:E13. 10.3171/2020.10.FOCUS20801 33386013 10.3171/2020.10.FOCUS20801

[33] Stienen MN, Abdulazim A, Gautschi OP, Schneiderhan TM, Hildebrandt G, Lucke S (2013) Calcifying pseudoneoplasms of the neuraxis (CAPNON): clinical features and therapeutic options. Acta Neurochir (Wien) 155:9–17. 10.1007/s00701-012-1502-2 23053277 10.1007/s00701-012-1502-2

[34] Stienen MN, Bellut D, Stojanov D, Eriks-Hoogland I, Regli L, Oertel MF (2019) Reversible Paraplegia - Favorable Outcome after Delayed Diagnosis. Praxis (Bern 1994) 108:341–345. 10.1024/1661-8157/a003205 30940039 10.1024/1661-8157/a003205

[35] Terrapon APR, Stienen MN, Veeravagu A, Fehlings M, Bozinov O, Hejrati N (2024) Intradural cystic schwannomas of the spine: a case-based systematic review of an unusual tumor. Brain Spine 4:102843. 10.1016/j.bas.2024.102843 38947985 10.1016/j.bas.2024.102843 PMC11214289

[36] Upadhyay J, Iwasaka-Neder J, Golden E, Bixby S (2023) Synthetic CT Assessment of Lesions in Children with Rare Musculoskeletal Diseases. Pediatrics 152:e2022061027. 10.1542/peds.2022-061027 37416976 10.1542/peds.2022-061027 PMC10389770

[37] Willesen ST, Hadsbjerg AE, Moller JM, Vladimirova N, Vora BMK, Seven S, Pedersen SJ, Ostergaard M (2024) MRI-based synthetic CT: a new method for structural damage assessment in the spine in patients with axial spondyloarthritis - a comparison with low-dose CT and radiography. Ann Rheum Dis 83:807–815. 10.1136/ard-2023-225444 38490729 10.1136/ard-2023-225444

[38] Yeo DK, Im SB, Park KW, Shin DS, Kim BT, Shin WH (2011) Profiles of spinal cord tumors removed through a unilateral hemilaminectomy. J Korean Neurosurg Soc 50:195–200. 10.3340/jkns.2011.50.3.195 22102948 10.3340/jkns.2011.50.3.195 PMC3218177

