# Supplementary material for: Evaluation of magnetic resonance imaging and deep learning-based synthetic computed tomography for calcified intradural tumors – importance of domain-specific training and validation of synthetic imaging methods for clinical application
Source: Acta Neurochir (Wien). 2025 Dec 11;168(1):1. doi: 10.1007/s00701-025-06731-0 (PMC12769552; doi:10.1007/s00701-025-06731-0)

Supplemental Figures

Supplemental Figure 1: Case of a 56-year-old female patient with myelopathy and an intradural tumor extending between the T5-7 levels. **A:** On conventional CT the tumor was visible, measuring 9 x 20mm with a mean density of 113 HUs. **B:** On synthetic CT the tumor was not visible, measuring 0 x 0mm with a mean density of 27.4 HUs. **C**: On close examination, the tumor is visible on the MRI source data. The patient underwent uneventful gross total resection via unilateral laminectomy of T6 (with removal of parts of T5 & T7). Histopathological workup revealed a WHO CNS grade-1 meningioma. The clinical & radiological follow-up until 12 months postoperative was uneventful.

A:
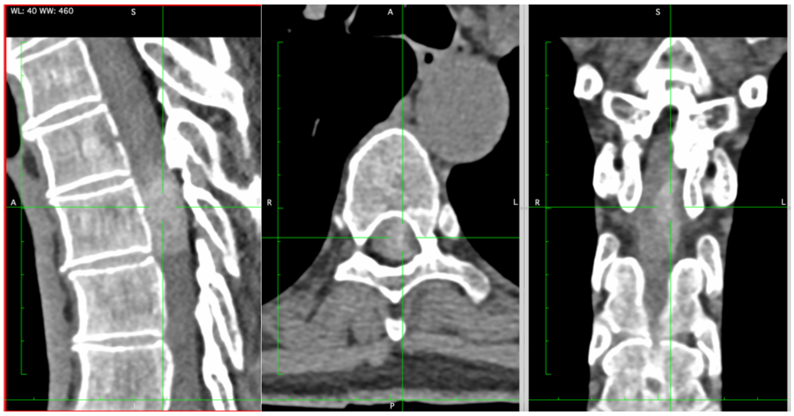

B:
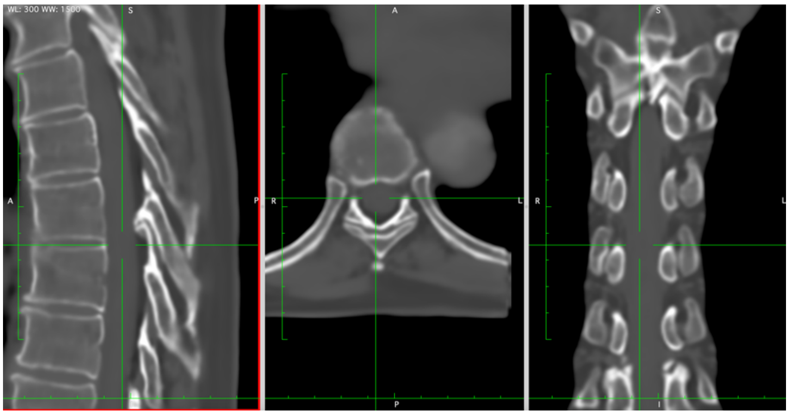

C:
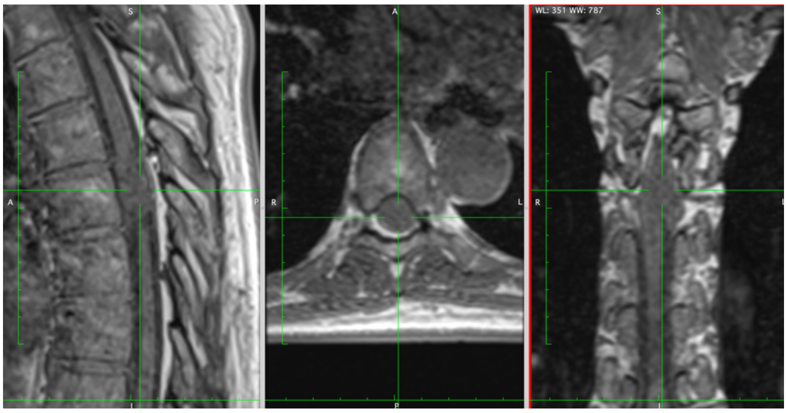

Supplemental Figure 2: Case of a 86-year-old neurologically intact male patient with an intradural tumor at the T4-5 level. **A:** On conventional CT the tumor was visible, measuring 11 x 8.1mm with a mean density of 265 HUs. **B:** On synthetic CT the tumor was not visible, measuring 0 x 0mm with a mean density of 16 HUs. **C**: The tumor is well visible on the MRI source data. The patient was managed conservatively with a stable clinical and radiological re-examination 12 months after the index imaging.

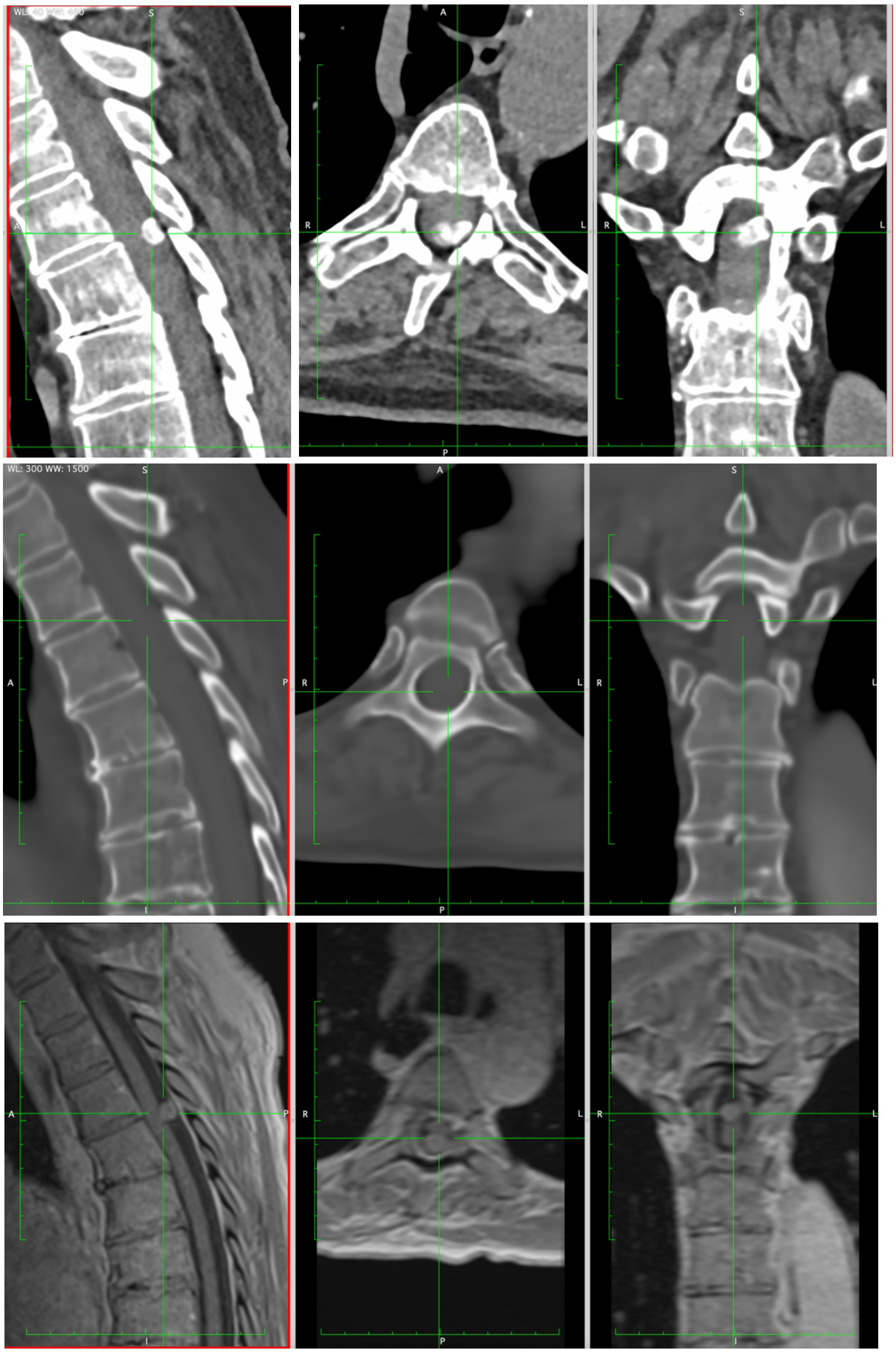

A:

B:

C:

Supplemental Figure 3: Case of a 55-year-old female patient with myelopathy and an intradural tumor extending between the T1-2 levels. **A:** On conventional CT the tumor was visible, measuring 9 x 16mm with a mean density of 407 HUs. **B:** On synthetic CT the tumor was not visible, measuring 0 x 0mm with a mean density of 23.9 HUs. **C**: The tumor is well-visible on the MRI source data. The patient underwent uneventful gross total resection via unilateral extensive fenestration of T1/2. Histopathological workup revealed a WHO CNS grade-1 meningioma. The clinical & radiological follow-up until 12 months postoperative was uneventful.

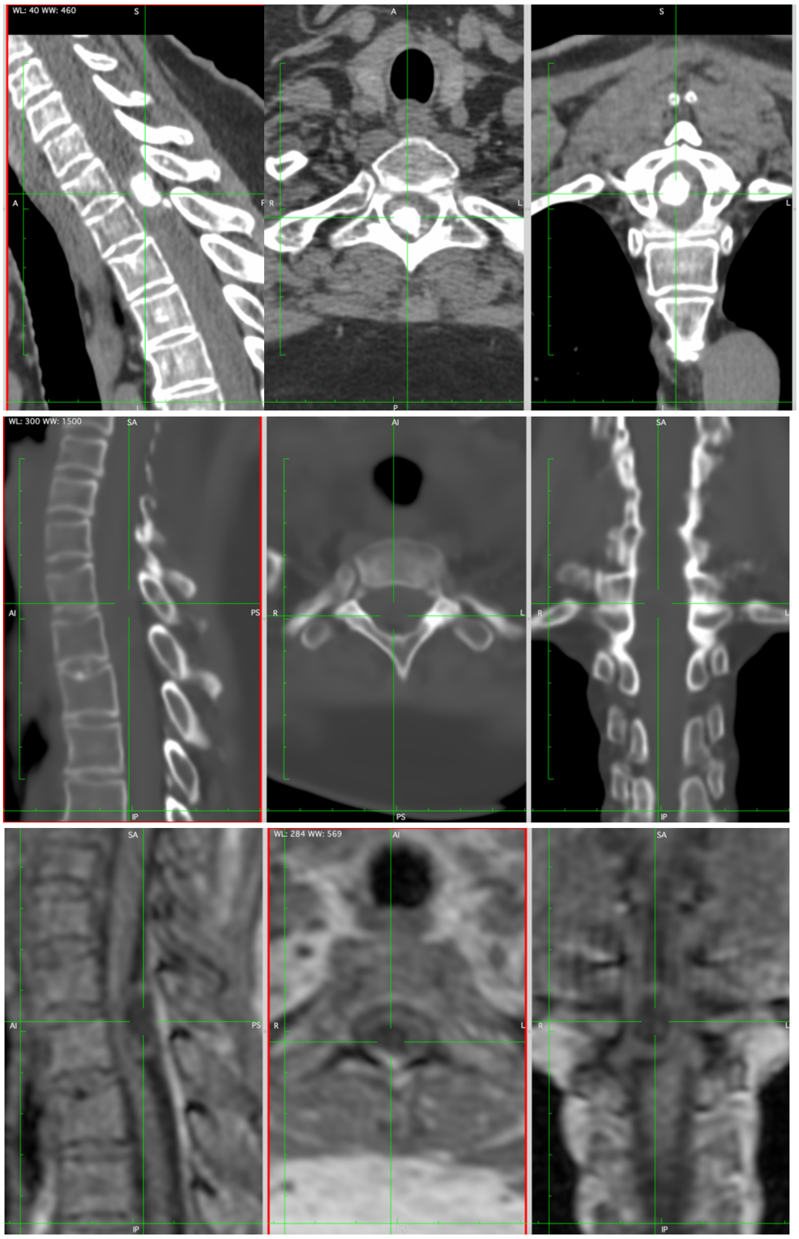

A:

B:

C:

Supplemental Figure 4: Case of a 71-year-old neurologically intact female patient with an intradural tumor extending between the C5-6 levels. **A:** On conventional CT the tumor was visible, measuring 13.8 x 16mm with a mean density of 628 HUs. **B:** On synthetic CT the tumor was not visible, measuring 0 x 0mm with a mean density of 14 HUs. **C**: The tumor is well-visible on the MRI source data. The patient underwent uneventful gross total resection via laminoplasty of C5-6. Histopathological workup revealed a WHO CNS grade-1 meningioma. The patient was hospitalized 15 days due to initially poorly controlled approach-related neck pain, but the remaining clinical & radiological follow-up until 12 months postoperative was uneventful.

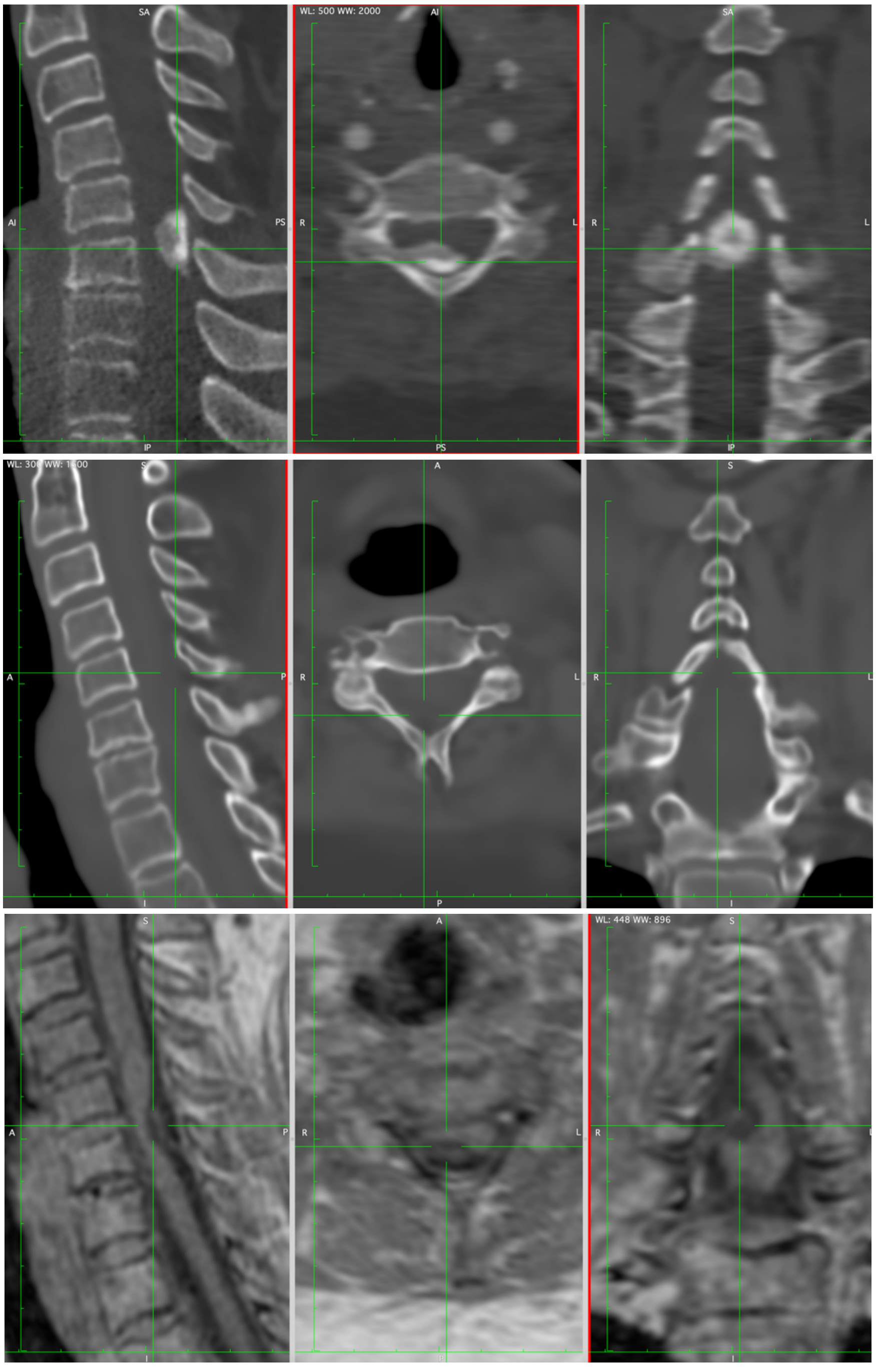

A:

B:

C:

Supplemental Figure 5: of a 68-year-old female patient (BMI 26.7kg/m^2) with a calcified, intradural tumorous lesion at the T8/9 level. The patient was myelopathic and presented with motor deficits of both lower extremities to our outpatient clinic. **A:** On conventional CT the tumor was visible, measuring 15 x 38mm with a mean density of 862.7 HUs. **B:** On synthetic CT the tumor was not visible, measuring 0 x 0mm with a mean density of 23.3 HUs. **C**: The tumor is well-visible on the MRI source data. The patient underwent uneventful gross total resection via laminoplasty of T8-9. Histopathological workup revealed a WHO CNS grade-1 meningioma. The clinical & radiological follow-up until 12 months postoperative was uneventful with gradual, almost complete neurological recovery.

A:

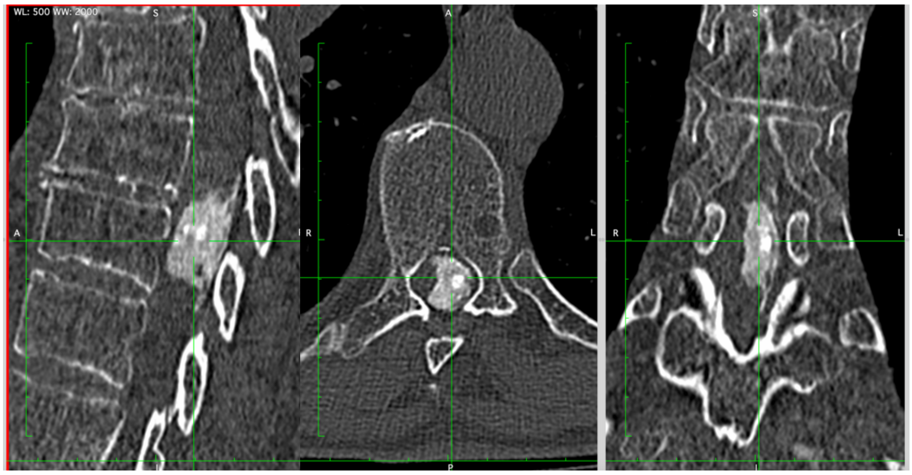

B:

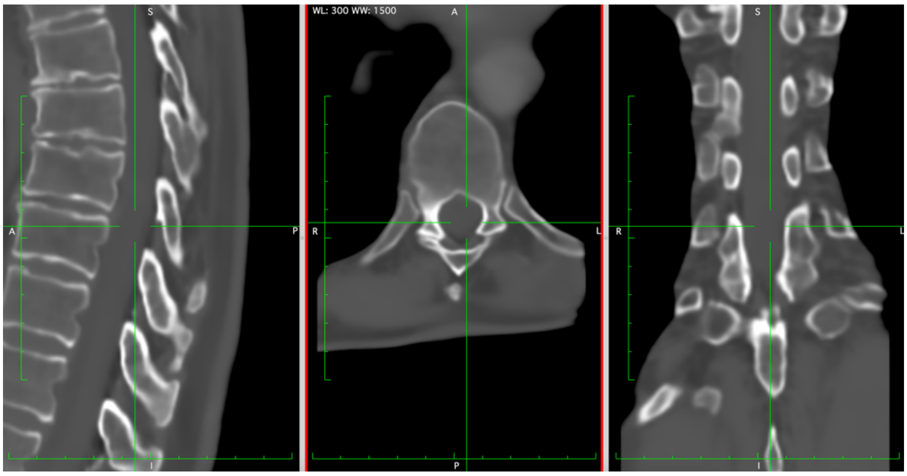

C:

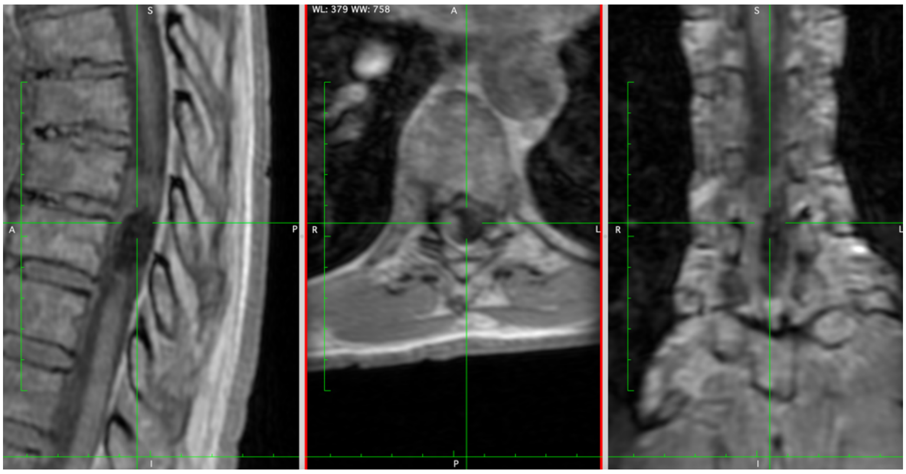

Supplement: Supplementary file 1 — Supplementary Material 1 (PDF 7.52 MB) [file 701_2025_6731_MOESM1_ESM.pdf]
